# Grazing effects of sea urchin *Diadema savignyi* on algal abundance and coral recruitment processes

**DOI:** 10.1038/s41598-020-77494-0

**Published:** 2020-11-23

**Authors:** Viet Do Hung Dang, Chia-Ling Fong, Jia-Ho Shiu, Yoko Nozawa

**Affiliations:** 1 Department of Life Science, National Taiwan Normal University, Taipei, 11677 Taiwan; 2 Biodiversity Program, Taiwan International Graduate Program, Academia Sinica and National Taiwan Normal University, Taipei, 11529 Taiwan; 3 Biodiversity Research Center, Academia Sinica, Taipei, 11529 Taiwan; 4 Institute of Marine Environment and Resources, Vietnam Academy of Science and Technology, Haiphong, 180000 Vietnam; 5 Graduate University of Sciences and Technology, Vietnam Academy of Science and Technology, Hanoi, 100000 Vietnam

**Keywords:** Ecology, Conservation biology, Restoration ecology, Tropical ecology

## Abstract

Herbivores control algae and promote coral dominance along coral reefs. However, the majority of previous studies have focused on herbivorous fish. Here we investigated grazing effects of the sea urchin *Diadema savignyi* on algal abundance and coral recruitment processes. We conducted an in situ cage experiment with three density conditions of *D. savignyi* (0, 8, 16 indiv. m^−2^) for three months during the main coral recruitment season in Taiwan. Results demonstrated a strong algal control by *D. savignyi*. At the end of the experiment, average algal cover was 95% for 0 indiv. m^−2^, compared to 47% for 8 indiv. m^−2^ and 16% for 16 indiv. m^−2^. Average algal biomass at 8 indiv. m^−2^ declined by one third compared to 0 indiv. m^−2^ and almost zero at 16 indiv. m^−2^. On the other hand, a negative grazing effect of *D. savignyi* was observed on coral recruitment processes. Notably, at 16 indiv. m^−2^, the density of coral recruits declined and mortality of small coral fragments (proxy of coral juveniles) increased. Our results confirm findings of previous studies and indicate the need to balance both positive (strong algal control) and negative (physical damage) influences of *Diadema* grazing to facilitate the coral recruitment process.

## Introduction

As the global decline in coral reefs becomes more evident^1–4^, the significance of our knowledge concerning factors contributing to coral resilience increases^5^. Traditionally, the interaction between three key functional groups; herbivores, algae and corals, is considered as a key component of coral recovery, in addition to other threats to coral reefs such as pollution, disease, and coral bleaching^1–5^. Herbivores control algae, algae compete with corals for space and corals help form an appropriate structure for herbivores to live on^6,7^. However, along many contemporary coral reefs, anthropogenic activities have distorted the healthy balance of these interactions (i.e., overfishing of herbivores and eutrophication favoring the proliferation of algae), which in turn hinder coral recovery after mortality events^7^. Therefore, ecological models on such interactions indicate that algal control is key to the recovery of coral populations via recovering herbivore assemblages and controlling land-based nutrient inputs^7,8^. Algal regulation via herbivory has been intensively studied in the Caribbean, along the Great Barrier Reef and in Kenya; mostly focusing on herbivorous fishes^5,7,9–14^. As a result, our current knowledge on algal control by herbivores is biased in terms of regions and taxa. Only a limited number of such studies originate from outside these regions, despite variation in coral reef ecosystems^15^. In particular, far less attention has been paid to non-fish herbivores, e.g., sea urchins and gastropods (except *Diadema antillarum* in the Caribbean).

To examine the effect of herbivores on algae, the majority of previous studies are in the form of exclusion cage experiments, where comparisons between ambient herbivory vs. non-herbivory conditions were observed^9,10,12,16–18^. While such experiments can provide useful presence/absence data, there are two main disadvantages in comparison to inclusion cage experiments. Firstly, the influence of non-target herbivore taxa cannot be well-controlled or documented. Secondly, this approach cannot examine a level of herbivory higher than the ambient level. This is necessary to understand optimal levels of herbivory to control algae, which is often higher than the ambient level on many overfished coral reefs today^9^.

*Diadema* is a cosmopolitan genus of sea urchin, widely distributed globally from temperate to tropical waters^19^. As *Diadema* species often form a dense population, they are considered ecologically important herbivores^20^. The best example is *D. antillarum,* that was dominant across the Caribbean pre-1980s, playing a central role in algal control^21^. The sudden decline of *D. antillarum* was the result of an outbreak of disease in 1983–1984, leading to algal blooms due to low functional redundancy of grazers; a consequence of overfishing herbivorous fishes. This hampered coral recovery and a phase shift from a coral-dominant to algal-dominant state occurred along many Caribbean reefs^21,22^. Interestingly, other *Diadema* species are still predominantly considered as noxious bioeroders that damage coral reefs by their destructive grazing activities^18,23–25^.

In our previous studies, we found that *Diadema* sea urchins were the dominant herbivore in southern Taiwan and that *Diadema* density positively correlated with juvenile coral density and coral recovery^26^. These findings motivated us to examine the effect of *Diadema* grazing on algal control and the coral recruitment process, as a primary driver of the observed correlations. Accordingly, in this study, we conducted an inclusion cage experiment, using the locally dominant *Diadema* species, *D. savignyi*, in southern Taiwan*.* Algal abundance, coral recruit density and growth and the survival of small coral fragments (proxy of coral juveniles) were examined under three density conditions of *D. savignyi* (0, 8, 16 indiv. m^−2^).

## Materials and methods

**Figure 1:**
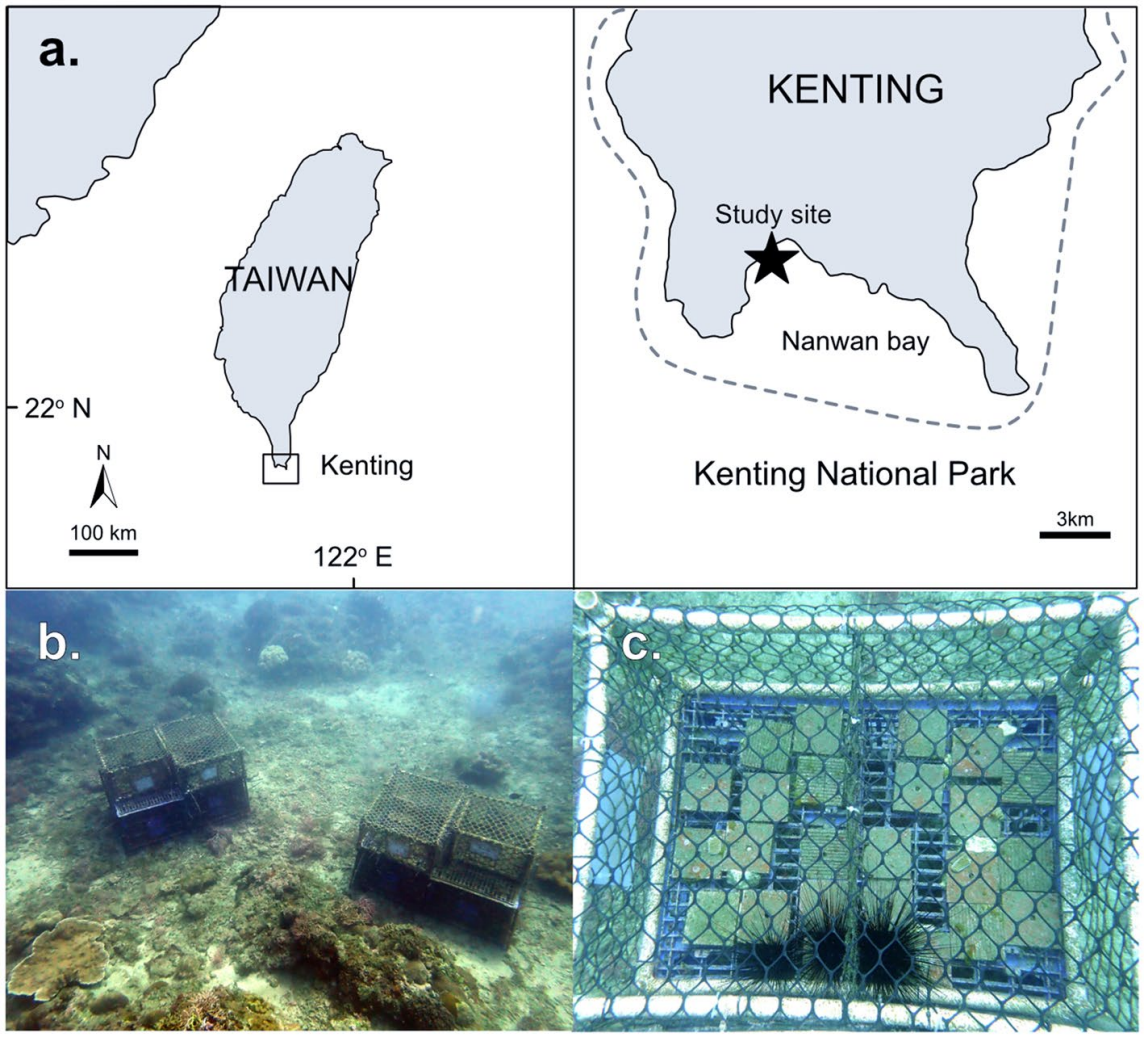
(**a**) Study location of the inclusion cage experiment at Nanwan bay, Kenting, Southern Taiwan. The area enclosed by the dashed line indicates Kenting National Park. The map was created using the package, maps ver. 3.3.0 with the software, R ver. 3.6.1 (https://www.rdocumentation.org/packages/maps/versions/3.3.0). (**b**,**c**) Image of the inclusion cage experiment: (**b**) two of the five experimental cage groups, each consisting of three cages for three *D. savignyi* density conditions on the top of plastic container base; (**c**) close-up of two cages with 8 indiv. m^−2^ (right) and 16 indiv. m^−2^ conditions (left) of *D. savignyi.* Each cage had 12 experimental terracotta plates (10 cm × 10 cm) fixed on the mesh bottom.

Cage experiments were conducted from March to June 2019 in Kenting, southern Taiwan (Fig. 1a). The study site was located in Kenting National Park where 235 species of hard coral (scleractinians) have been documented^27^. The study area was at 12 m depth, consisting of hard substrata and a sandy bottom on which experimental cages were affixed (Fig. 1b). The experimental period covered the major coral recruitment season (March to June) estimated at the study location^28^. Seawater temperature was recorded using Hobo data-loggers (HOBO Pendant Temperature/Light 64 K Data Logger; Onset Computer Corporation, Bourne, MA, USA).

In the cage experiment, we used three density conditions of sea urchins (0, 8, 16 indiv. m^−2^) and examined algal abundance, coral recruitment and growth and the survival of small coral fragments that were used as a proxy for coral juveniles. Experimental cages (L × W × H: 60 × 40 × 25 cm) were created using PVC pipes and plastic mesh (mesh hole size of 5 cm) (Fig. 1c). Three experimental cages corresponding to the three density conditions were grouped and fixed on a foundation made from plastic containers (L × W × H: 120 × 80 × 50 cm) that were attached on the sandy bottom using 1-m iron pegs (Fig. 1b). Five groups of experimental cages were established as replicates at a distance of ca. 2–5 m from each other.

Twelve terracotta plates (10 cm × 10 cm) were fixed on the bottom of each experimental cage using underwater epoxy; three plates for algae, six plates for coral recruits and three plates for coral fragments (Fig. 1c). For algae and coral fragments, plates with plane surfaces were used. For coral recruits, plates with plane and grooved surfaces (14 grooves per surface; groove size: 4 mm wide × 100 mm long × 2 mm deep) were paired and used to examine the effect of surface structure on coral recruits under sea urchin grazing^11,29^. All plates and plate pairs were randomly deployed on the cage bottom. Three coral species were selected for coral fragments: fast-growing and branching coral *Acropora solitaryensis*, fast-growing and encrusting coral *Montipora undata*, and slow-growing massive coral *Porites* sp.^30^. For each species, three small fragments (ca. 2–3 cm in diameter) were collected from five different donor colonies at the study site and introduced into the three density condition cages in each of the five groups (i.e., n = 5 fragments from different colonies per density condition). Coral fragments were fixed on separate plates using super glue.

Prior to the experiment, cages were setup and maintained at the study site for two months (January and February 2019) without sea urchins, to biologically condition terracotta plates for coral recruits and gain some algae on plate surfaces for initial food for sea urchins. When the experiment commenced in March, sea urchins were introduced into cages which were then sealed with plastic mesh. Locally dominant sea urchins *D. savignyi* were collected at the study site (the test diameter of 5.2–6.3 cm) and used in the experiment. Three experimental cages in each cage group were randomly allocated for the three density conditions. Zero, one and two *D. savignyi* individuals were then introduced into each experimental cage. As the bottom of experimental cages were plastic mesh and had many gaps (Fig. 1c), we considered the total surface area of 12 plates (0.12 m^2^) as a hard substrate where *D. savignyi* could mainly graze in each cage. Therefore, for zero, one, and two *D. savignyi* conditions, we calculated the *D. savignyi* density as approximately 0, 8, and 16 indiv. m^−2^, respectively. Sea urchin density was monitored and experimental cages were cleaned each month during the experimental period. When any sea urchin escaped or died, it was replaced with a new one (Supplementary Table S1).

In each experimental cage, algal cover (macroalgae and turf algae) on three algal plates and the survival and growth of coral fragments were monitored monthly by photographic images. Total algal cover (%) on the three algal plates was estimated by the CPCe method, with 50 random points overlaid per plate using the CPCe v 4.0 software^31^. Growth of each fragment was estimated by measuring the planar increment (or decrement) of coral tissue area from photographic images using ImageJ v1.31 software^32^. The relative growth rate of each fragment was calculated by dividing the increased (or decreased) tissue area over the 3-month experimental period by the initial tissue area.

At the end of the 3-month experiment, we collected three algal plates and three plate pairs (plane and grooved) for coral recruits from each experimental cage to examine algal biomass (dry weight) and coral recruit density in the laboratory. After gently rinsing with tap water to remove sand and sediment, algae on each plate were dried in an oven at 60 °C for over 48 h, scraped off and weighed on an electric scale. The sum of algal dry weight from the three algal plates was used for the analysis. Plates for coral recruits were soaked in a 10% bleach solution for 48 h to remove soft-tissue benthos from the plate surface, dried under sunlight, and examined for coral recruits under a stereomicroscope. Coral recruits were identified to the family level according to Babcock et al.^33^.

To deal with non-normality of data and the randomized block design in the experiment, we used generalized linear models (GLMs) to examine the effect of *D. savignyi* density on algal cover, algal biomass, coral recruit density and growth of coral fragments. Cage groups and plates on which algal cover was monitored (monthly) were initially considered as random factors, but treated as fixed factors in the GLMs because of the low replication number (n = 5)^34^. In GLMs for algal cover, binomial distribution with the logit link function was used for the original CPCe data, where the number of random points overlapped with algae was counted on the photographic image. Sea urchin density and time (month) after the start of experiment were incorporated as fixed factors. In GLMs for algal biomass, gamma distribution with the inverse link function were used. In GLMs for coral recruits, poisson distribution and the log link function were used. Coral recruits on plane surface plates were analyzed with a zero-inflated poisson model to deal with an excess zero counts in the data. For the GLM for growth of coral fragments, a gamma distribution with the inverse link function were used for a created variable of absolute growth per unit area (value range − 1 to ∞), plus one to cancel out negative values for a gamma distribution. For these GLMs, sea urchin density and cage groups were incorporated as fixed factors. For coral recruits on grooved surface plates, coral taxa were additionally incorporated as a fixed factor in the GLM, to examine the interaction effect with sea urchin density. Overdispersion in the binomial and poisson GLMs was fixed using the quasi-likelihood approach^35^. The GLMs were analyzed by likelihood ratio tests to examine any statistical significance (*p* < 0.05) of fixed factors. When a significance was detected, a post-hoc test (Turkey’s HSD) based on the fitted GLM was conducted for pairwise comparisons. All statistical analyses were done in R ver. 3.6.1 software^36^, with the glm function of lme4 ver. 1.1-21 package for constructing GLMs, the glm.binomial.disp function of dispmod ver. 1.2 package for fixing overdispersion, the zeroinfl function of pscl ver. 1.5.5 package for constructing the zero-inflated poisson model, the Anova function of car ver. 3.0-7 package for the test of deviance, and the glht function of multcomp ver. 1.4-13 package for the post-hoc test.

## Results

During the 3-month experiment, the weather was calm and seawater temperature was 26.9 ± 1.2 °C (mean ± SD). There were two sea urchins that died, two escaped and one moved into another cage due to a mesh breakage (Supplementary Table S1). These cases happened once in two cages and twice in two cages. In each case, the sea urchin density was restored to the original density within 1 month.

**Figure 2:**
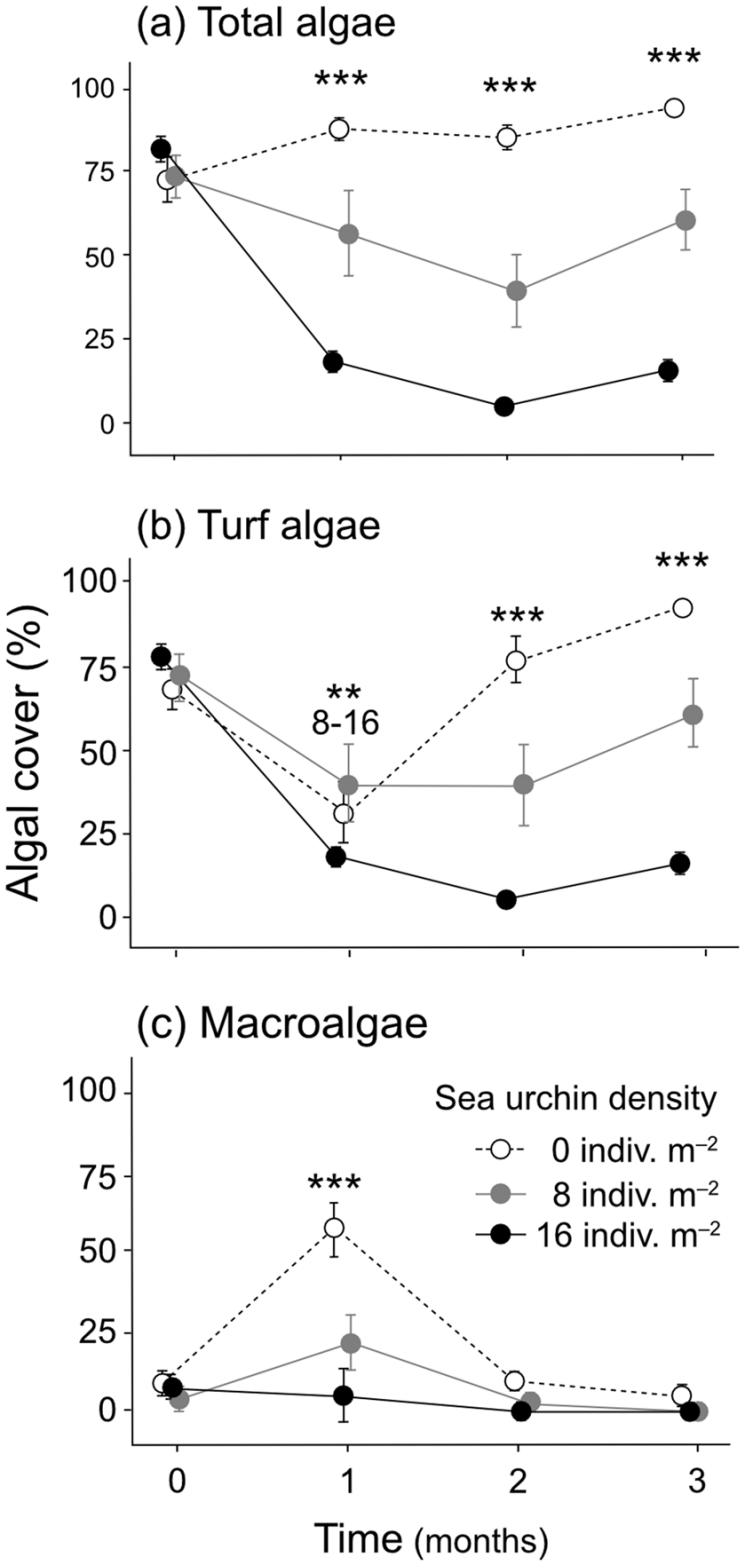
The effect of three density conditions of *Diadema savignyi* on algal cover during the 3-month inclusion cage experiment. Average ± SE are shown. Stars indicate statistical significance at *p* < 0.01 (**) and *p* < 0.001 (***); (**a**) between the three density conditions from the 1st to 3rd month, (**b**) between 8 and 16 indiv. m^−2^ at the 1st month and between the three density conditions at the 2nd and 3rd month, and (**c**) between the three density conditions at the 1st month. Datapoints were slightly shifted horizontally to avoid overlapping.

Total algal cover changed among the three density conditions of *D. savignyi* (Tukey's HSD, *p* < 0.001; Fig. 2a). In the 0 indiv. m^−2^ condition, algal cover slightly increased from initially 73 to 95% on average over the 3-month experimental period. For the 8 indiv. m^−2^ condition, average algal cover declined gradually from an initial 73 to 40% by the 2nd month and remained at 47% by the 3rd month. In the 16 indiv. m^−2^ condition, average algal cover dropped from 82 to 20% by the 1st month and remained at a low cover from thereon; 5% in the 2nd month and 16% in the 3rd month. When the total algal cover was separated into two algal types, turf algae and macroalgae, turf algae dominated plate surfaces (Fig. 2b), while the seasonal peak of macroalgae, mainly *Colpomenia* sp. was observed once in the 1st month (April) in the 0 indiv. m^−2^ condition (57% on average) and the 8 indiv. m^−2^ condition (17%) (Tukey's HSD, *p* < 0.001, respectively; Fig. 2c, Supplementary Fig. S1).

**Figure 3:**
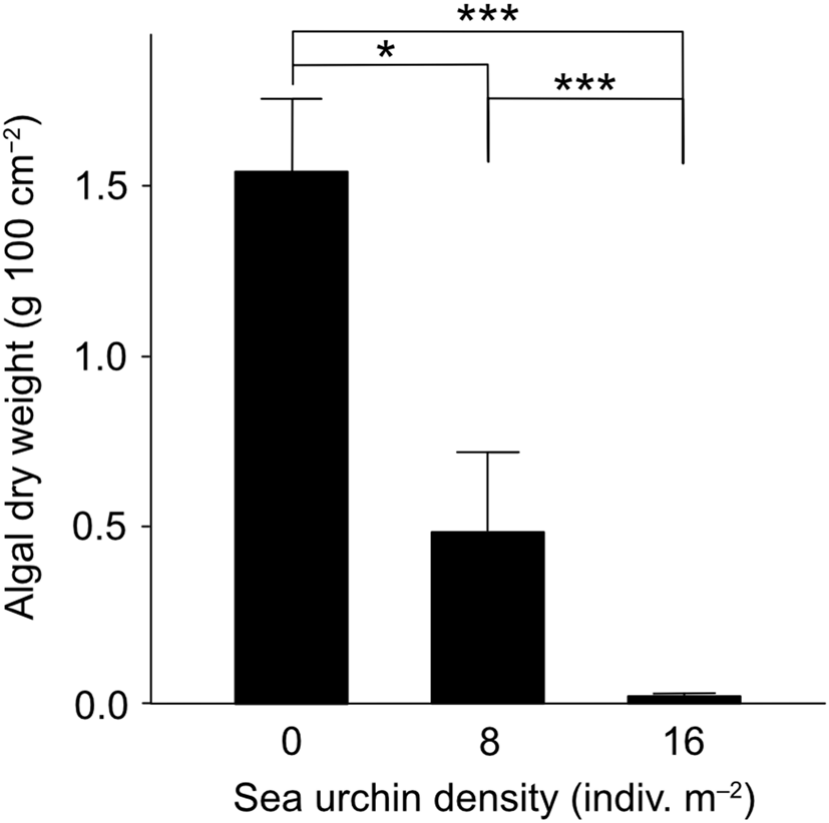
The effect of three density conditions of *Diadema savignyi* on algal biomass at the end of the 3-month experiment. Average + SE are shown. Stars denote statistical significance at *p* < 0.05 (*) and *p* < 0.001 (***).

Algal biomass at the end of the 3-month experiment was significantly lower at higher density conditions of *D. savignyi* (Fig. 3). The average algal biomass in the 8 indiv. m^−2^ condition (0.5 g 100 cm^−2^) was one third of that in the 0 indiv. m^−2^ condition (1.5) and was close to zero in the 16 indiv. m^−2^ condition (0.02).

**Figure 4:**
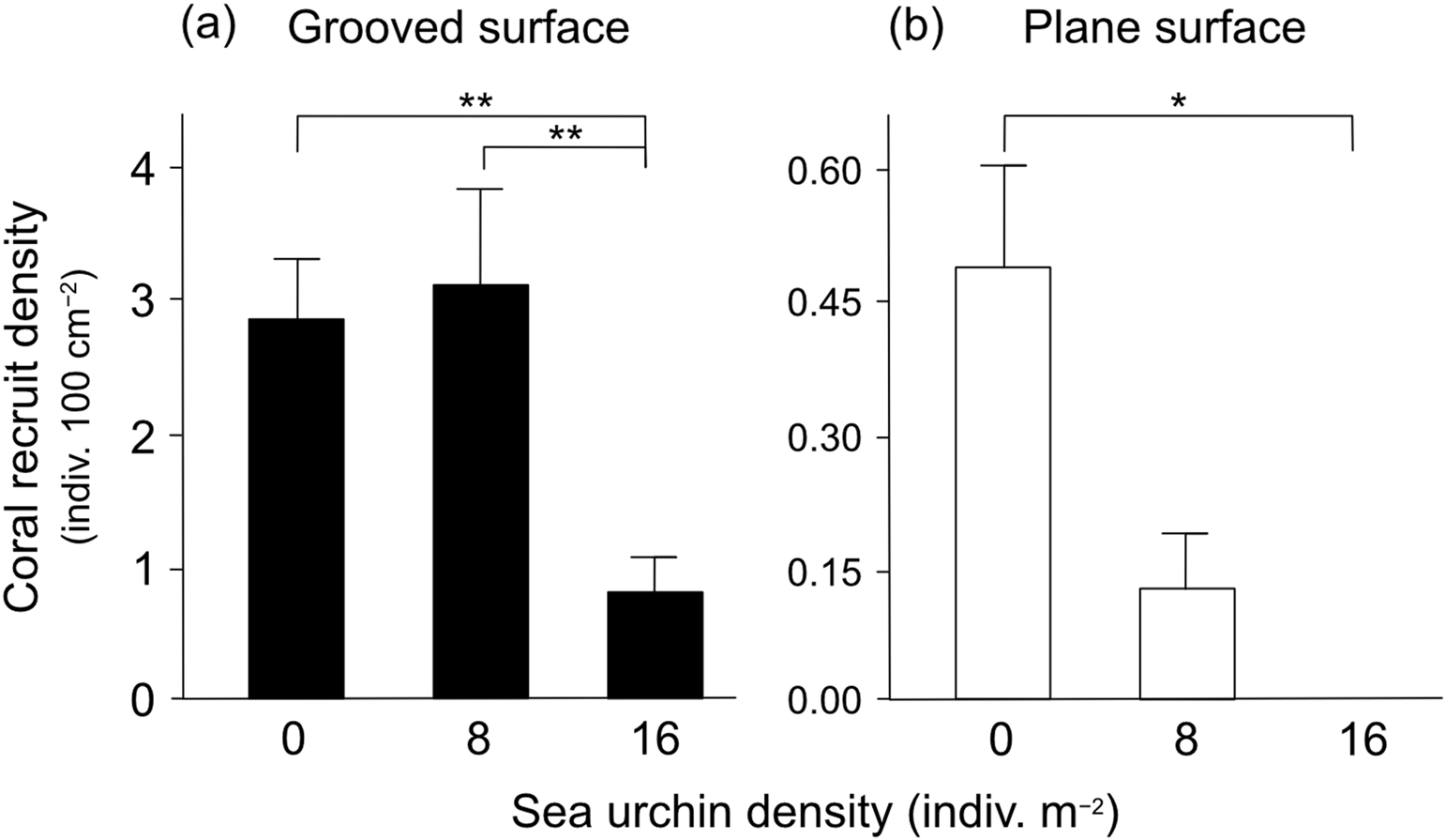
The effect of the three density conditions of *Diadema savignyi* on coral recruitment density (**a**) on grooved surface plates and (**b**) plane surface plates at the end of 3-month experiment. Average + SE are shown. Stars denote statistical significance at *p* < 0.05 (*) and *p* < 0.01 (**). No coral recruits occurred on plane plates in the 16 indiv. m^−2^ condition.

A total of 101 coral recruits were recorded on plates with grooved surfaces, whereas only 9 coral recruits were found on plates with plane surfaces at the end of the 3-month experiment. On grooved surfaces, coral recruit density at 0 indiv. m^−2^ (average: 2.9 indiv. 100 cm^−2^) and 8 indiv. m^−2^ (3.1 indiv. 100 cm^−2^) were similar and significantly higher than that in 16 indiv. m^−2^ (0.8 indiv. 100 cm^−2^; Turkey’s HSD, *p* < 0.01) (Fig. 4a). The dominant family of coral recruits was Poritidae (53.6%), followed by Pocilloporidae (27.3%). No interaction effect was detected between the sea urchin density conditions and the coral recruit taxa (likelihood ratio test, *p* = 0.92). Most coral recruits were observed within the grooves of plates, irrespective of density of *D. savignyi*. On plane surfaces, coral recruit densities were much lower and appeared to be more strongly affected by the *D. savignyi* density condition compared with that of grooved surfaces (Fig. 4b). The highest average density of coral recruits was recorded at 0 indiv. m^−2^ (0.47 indiv. 100 cm^−2^), followed by 8 indiv. m^−2^ (0.13 indiv. 100 cm^−2^), although this difference was not statistically significant (Turkey’s HSD, *p* > 0.05). In 16 indiv. m^−2^, no coral recruits were observed.

During the 3-month experiment, all fragments survived in the 0 indiv. m^−2^ condition, except for one fragment of *A. solitaryensis*. In the 8 indiv. m^−2^ condition, fragments of *M. undata* were affected most and all fragments died by the 3rd month. In the 16 indiv. m^−2^ condition, all fragments of *M. undata* died by the 1st month and three of five fragments of *A. solitaryensis* died by the end of 3-month experiment, whereas four of five *Porites* fragments still survived at the 3rd month. Mortality of coral fragments are summarized in Table 1.

**Table 1:** Coral fragment mortality.

Sea urchin density	1st M	2nd M	3rd M
*Acropora solitaryensis*			
0	0	0	1
8	0	0	0
16	1	3	3
*Montipora undata*			
0	0	0	0
8	0	2	5
16	5	−	−
Massive *Porites* sp.			
0	0	0	0
8	0	0	1
16	0	1	1

The table presents the cumulative number of dead coral fragments (n = 5 replicates) in each *Diadema savignyi* density condition (indiv. m^−2^) by the 1st, 2nd and 3rd month observations. Horizontal bars in *M. undata* indicate the termination of observation due to 100% mortality by the 1st month.

Growth of coral fragments at the end of the 3-month experiment were compared between the density conditions of *D. savignyi* for each species (Supplementary Fig. S2). Due to the high mortality, growth rates of *M. undata* and *A. solitaryensis* fragments in 16 indiv. m^−2^ were not included in the analysis. For *A. solitaryensis*, no significant difference was found between 0 and 8 indiv. m^−2^ (Turkey’s HSD, *p* = 0.72). For massive *Porites* sp., a significantly higher growth rate was detected in 8 indiv. m^−2^ compared to 16 indiv. m^−2^ (Turkey’s HSD, *p* < 0.05).

## Discussion

This study investigated the grazing effects of the sea urchin *D. savignyi* on algal abundance and coral recruitment processes using an inclusion cage experiment. Results demonstrated strong algal control by *D. savignyi*. On the other hand, coral recruitment processes were clearly negatively influenced at the highest density condition (16 indiv. m^−2^), with lower recruitment rates and higher mortality of small coral fragments (proxy for coral juveniles). While the strong algal control would facilitate the coral recruitment process and aid coral dominance, the negative impact of destructive grazing by *D. savignyi* supports previous studies^11,18,37,38^, and indicates the need for density control when considered for coral reef management.

### Effect of *Diadema* grazing on algal control

The majority of information regarding the effects of sea urchin grazing on algal control along coral reef ecosystems comes from *D. antillarum* in the Caribbean^11,39–44^. This is because historically, *D. antillarum* was the dominant herbivore in the Caribbean due to overfishing of other macro-herbivores^45^. Elimination and exclusion studies of *D. antillarum* demonstrated the strong algal control by *D. antillarum*, with significant increases in algal abundance within several months after the start of the experiment^11,39–41,43^. Subsequent die-offs of *D. antillarum* in 1983–1984 caused an algal-dominant state post-1983 on many Caribbean coral reefs, which further highlighted its important role in algal control along the reefs^21,22^. For other *Diadema* species, only some limited information is available for *D. setosum* and *D. savignyi* in Kenya^13,18,46,47^ and *D. setosum* in the Red Sea^48–50^. Although these studies examined the effects of *Diadema* grazing together with other coinhabiting herbivores in the field, the results generally support the strong capability of algal control by *Diadema* species.

While the effect of *Diadema* grazing on algal control has become apparent, the effective density of *Diadema* on algal control has not been well-explored. Such information would be best examined by in situ sea urchin inclusion cage experiments^9^. However, only a limited number of inclusion cage studies of sea urchins have been done for coral reef ecosystems^11,41,43^. Sammarco^11,41^ examined four densities of *D. antillarum* (0, 4, 16, 64 indiv. m^−2^) at Discovery Bay, Jamaica. The author observed that algal biomass at the two highest densities was nearly zero, whereas at 4 indiv. m^−2^ was similar to that at the 0 indiv. m^−2^. Carpenter^43^ examined the effect of *D. antillarum* grazing at a density of 4.9 indiv. m^−2^ at St. Croix, Virgin Islands. He found that the density significantly reduced algal biomass to approximately one quarter of that in the herbivore exclusion condition. In the present study, *D. savignyi* densities of 8 and 16 indiv. m^−2^ opened free space of approximately 50% and 80%, reducing algal biomass to one third of that in 0 indiv. m^−2^ and nearly zero, respectivly. Although not in a coral reef area, Ishikawa et al.^51^ examined five densities of *D. setosum* (0, 1, 2, 4, 8 indiv. m^−2^) at Haidaura Bay, central Japan (33°N). Results of algal cover indicated approximately a 50% reduction in 1 and 2 indiv. m^−2^ conditions and nearly a 100% reduction in 4 and 8 indiv. m^−2^ conditions. Algal biomass was reduced to one tenth in 1 and 2 indiv. m^−2^ conditions and nearly zero in 4 and 8 indiv. m^−2^ conditions. As seen in these four studies, the results indicate a high variation in effective *Diadema* densities on algal control among cases. This is likely owing to differences in local conditions (e.g., nutritional and physical environment and algal assemblage) and examined *Diadema* species, suggesting that it may be necessary to examine effective *Diadema* density at each location, which may also vary with time as local conditions change.

### Effects of *Diadema* grazing on coral recruitment process

Sea urchin grazing exerts both positive and negative influences on coral recruitment processes, via the removal of algal competitors and its destructive nature^11,18,37,52,53^. Therefore, the gross effect of sea urchin grazing on coral recruitment processes should be determined by its net positive and net negative effects, which may vary depending on sea urchin species and density, coral taxa, and structural complexity of substrate surface^11,18,37,52,54–56^.

Regarding the effect of *Diadema* density on the coral recruitment process, information is scarce due to a lack of density manipulation experiments. Specifically, for *Diadema* sea urchins, Sammarco^11^ was the only study, besides the present study, that examined the effect of density on coral recruitment processes. The author found optimal conditions for survival and growth of coral recruits at the intermediate densities of *D. antillarum*, especially at 4 indiv. m^−2^. In the present study, the negative effect of *D. savignyi* grazing was limited in the 8 indiv. m^−2^ condition, while *D. savignyi* grazing was clearly detrimental in the 16 indiv. m^−2^ condition. Field observational studies have reported that populations of *D. antillarum* (1.7–8.9 indiv. m^−2^) in the Caribbean created algal free zones where the density of juvenile corals was up to 11-fold higher than surrounding algal zones^44,56,57^. Collectively, the current limited information suggests that, while causing some negative impacts, the maximum *Diadema* density of ≤  ~ 8 indiv. m^−2^ would benefit coral recruitment processes, controlling algae and enhancing coral juvenile density.

There is some information available on species-specific responses of coral recruits to *Diadema* grazing, mostly on *D. antillarum* in the Caribbean^11,53,56,57^. Sammarco^11^ found that, among three dominant genera of coral juveniles, *Favia fragum* were most susceptible, with rapidly decreasing density in higher *D. antillarum* densities in comparison with *Agaricia* spp. and *Porites* spp. Carpenter and Edmunds^56^ reported that recovered populations of *D. antillarum* (1.7–8.9 indiv. m^−2^) were associated with high densities of juvenile corals, mostly *Agaricia* spp. and *Porites* spp., but were rarely associated with certain coral genera, e.g. *Acropora* and *Montastraea*, resulting in the reduction of generic diversity of coral juveniles in *D. antillarum* zones. Idjadi et al.^57^ recorded the highest growth rates of juvenile corals in *Porites* spp. in the recovered population of *D. antillarum* (2.7–4.1 indiv. m^−2^) in Jamaica. Davies et al.^53^ observed that growth rates of *Agaricia* recruits declined, whereas that of *Porites* recruits did not change in *D. antillarum* inclusion cages. In the present study, *M. undata* was the most susceptible with no survivors in *D. savignyi* inclusion cages, followed by *A. solitaryensis* that survived only in 8 indiv. m^−2^, whereas massive *Porites* sp. mostly survived. These results demonstrate inter-species variation in the effects of *Diadema* grazing during the early life-history stages of corals. Recruits and juveniles of some coral taxa, e.g. *Porites*, appear to thrive under *Diadema* grazing, while others, e.g. *F. fragum*, performed better without *Diadema* grazing, coinhabiting with algae. The inter-species variation may relate to their life-history strategies, e.g. growth-speed to attain refuge size from grazing damage and a tolerance to algal competitors^37,58,59^. In nature, the negative effect of *Diadema* grazing could be mitigated by substrate complexity^11,29^ and variation in densities of *Diadema* sea urchins that create a gradient of *Diadema* grazing intensity along habitats.

Previous studies found that complex surface structure of substrata is important in providing refuge space for coral recruits from the destructive grazing of herbivores, enhancing their survivorship, while benefiting from the removal of algal competitors^11,29,37,60^. The results of the present study confirm this finding, where coral recruits mostly occurred in the grooves of plates in *Diadema* inclusion cages. Interestingly, the groove structure (4 mm wide and 2 mm deep) appeared not to be an effective refuge against *Diadema* grazing at 16 indiv. m^−2^, resulting in the decline of overall coral recruitment. Although the number of coral recruits on plane plates was much lower compared to grooved plates even at 0 indiv. m^−2^, this could be explained by the occurrence of shaded areas on the grooved plate surface, created by the grooves where less algae grew and most coral recruits occurred. Whereas the plane plate surface, which was mostly covered by algae, prevented settlement of coral larvae^12^.

## Conclusions

The present study confirms the findings of previous studies and elucidates both the positive (strong algal control) and negative (physical damage) influence of *Diadema* grazing on the coral recruitment process. As the positive influence of algal removal would be simply a function of *Diadema* density, we may need to focus more on the *Diadema* density that minimizes the negative influence of *Diadema* grazing to determine the optimal density for the coral recruitment process. To this end, future studies should explore the effective *Diadema* density in a lower density range (i.e. < 4–8 indiv. m^−2^) than the present and previous studies employed or reported. The available information suggests that the optimal *Diadema* density would vary among locations and probably time, depending on environmental conditions (e.g., nutrition, algal taxa, sedimentation and temperature). Therefore, when considering the use of *Diadema* grazing for algal control to promote coral recruitment, careful assessment of densities needs to be involved in management decisions to ensure that the optimal density of *Diadema* is maintained. Promising results have been reported from initial studies that employed sea urchin grazing to control algae on coral reefs in Jamaica and Hawaii^61,62^.

## Supplementary information

Supplementary Information.
