# Supplementary material for: Grazing effects of sea urchin Diadema savignyi on algal abundance and coral recruitment processes
Source: Sci Rep. 2020 Nov 23;10:20346. doi: 10.1038/s41598-020-77494-0 (PMC7684293; doi:10.1038/s41598-020-77494-0)
Supplement: Supplementary file 1 — Supplementary Information. [file 41598_2020_77494_MOESM1_ESM.docx]

**SUPPORTING INFORMATION**

**“Grazing effects of sea urchin *Diadema savignyi* on algal abundance and coral recruitment processes”** by Viet Do Hung Dang, Chia-Ling Fong, Jia-Ho Shiu and Yoko Nozawa

**Supplementary Table S1.** The number of *Diadema savignyi* individuals that escaped, died or moved into another cage recorded by monthly monitoring. Cage ID consists of grouping ID (A–E) and sea urchin density condition (0, 8, 16 indiv. m^-2^). The experimental density condition was recovered within one month at each monthly monitoring.

| **Cage ID** | | **Month** | | |
| --- | --- | --- | --- | --- |
| Group | Urchin density | *1st* | *2nd* | *3rd* |
| A | 0 |  |  |  |
| A | 8 |  | 1 escaped |  |
| A | 16 |  |  |  |
| B | 0 |  |  |  |
| B | 8 |  |  |  |
| B | 16 |  |  |  |
| C | 0 |  |  |  |
| C | 8 |  |  |  |
| C | 16 |  |  |  |
| D | 0 |  |  |  |
| D | 8 |  |  | 1 died |
| D | 16 |  |  |  |
| E | 0 |  |  |  |
| E | 8 |  | 1 escaped | 1 moved from E16 |
| E | 16 | 1 died |  | 1 moved to E8 |


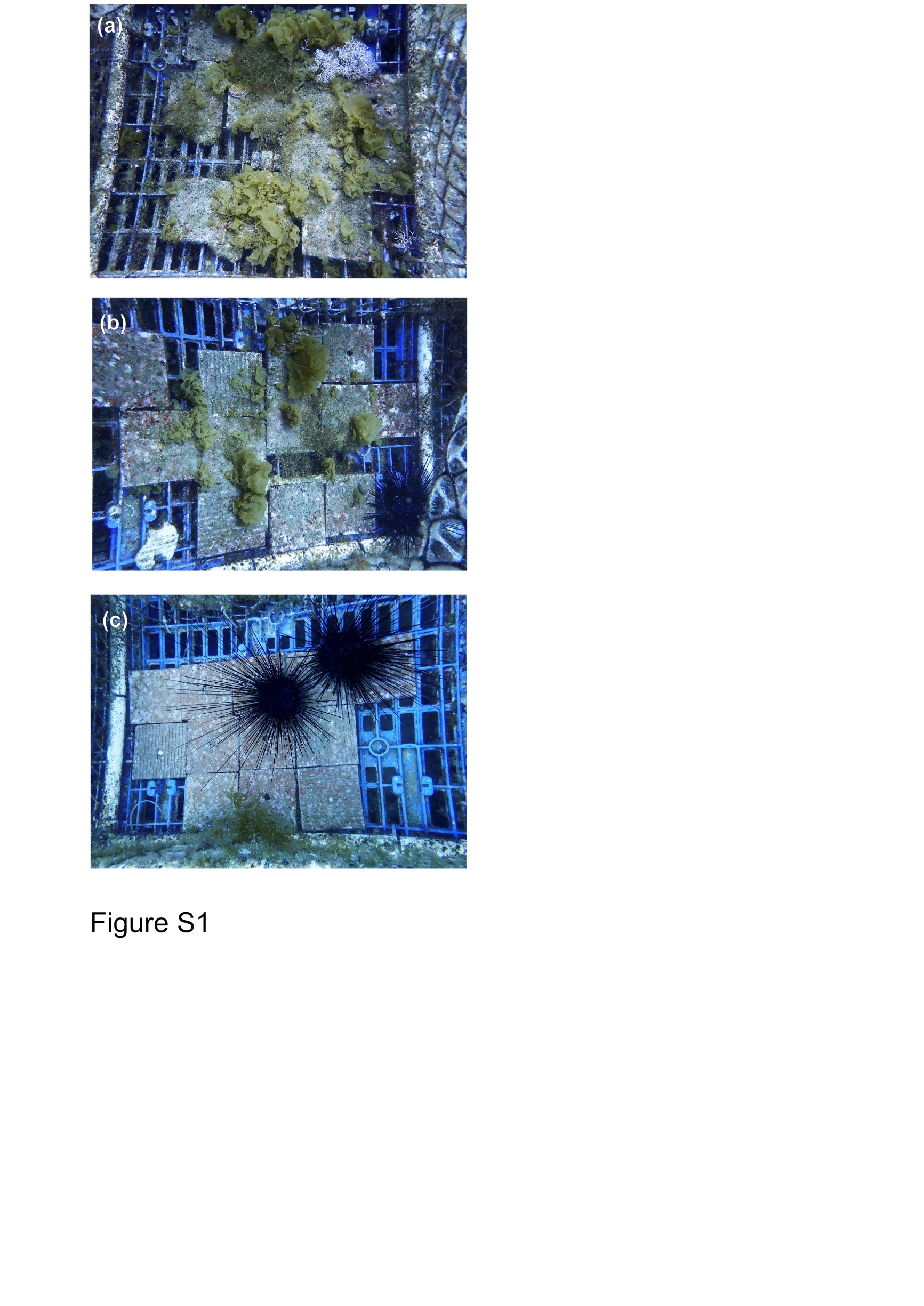


**Supplementary Fig. S1.** Images of the *Diadema savignyi* inclusion cage at the 1st month of the experiment: (a) 0 individual m^-2^ condition with 0 sea urchin; (b) 8 individual m^-2^ condition with 1 sea urchin; and (c) 16 individual m^-2^ condition with 2 sea urchins.


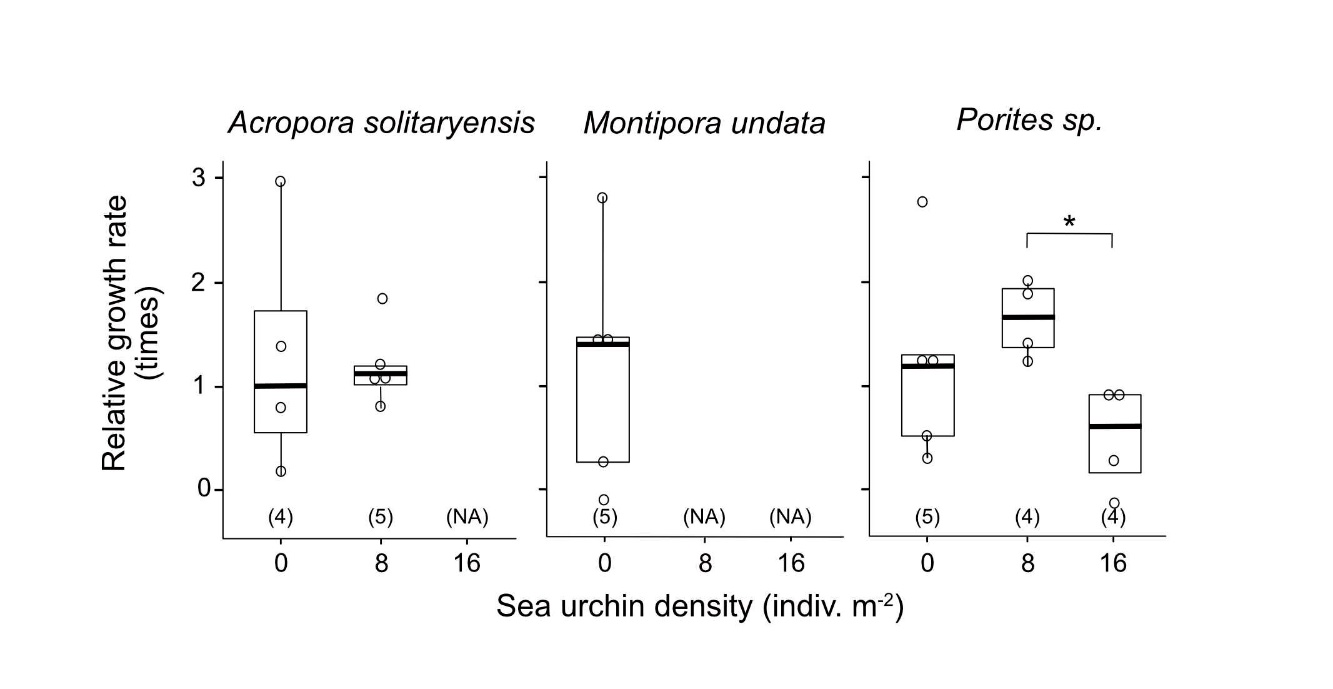


**Supplementary Fig. S2.** Growth rates of coral fragments at the 3^rd^ month of the cage experiment. Fragments from three coral species were examined under the three density conditions of *Diadema savignyi* (0, 8, 16 indiv. m^-2^). Data indicates relative growth rates (ratio of increment, or decrement area to initial area in the 3-month experiment). The number in brackets indicates the number of surviving fragments at the 3^rd^ month of the experiment used for the estimate. “NA” denotes that data is not available for the growth estimate due to 100% mortality or only two fragments surviving in *A. solitaryensis*. The star mark indicates a statistical significance of p < 0.05.
